# Perspective: Disentangling the effects of tES on neurovascular unit

**DOI:** 10.3389/fneur.2022.1038700

**Published:** 2023-01-09

**Authors:** Yashika Arora, Anirban Dutta

**Affiliations:** ^1^Neuroimaging and Neurospectroscopy (NINS) Laboratory, National Brain Research Centre, Gurugram, India; ^2^School of Engineering, University of Lincoln, Lincoln, United Kingdom

**Keywords:** transcranial electrical stimulation, functional MRI (fMRI), computational modeling, systems biology, model predictive control

## Abstract

Transcranial electrical stimulation (tES) can modulate the neurovascular unit, including the perivascular space morphology, but the mechanisms are unclear. In this perspective article, we used an open-source “rsHRF toolbox” and an open-source functional magnetic resonance imaging (fMRI) transcranial direct current stimulation (tDCS) data set to show the effects of tDCS on the temporal profile of the haemodynamic response function (HRF). We investigated the effects of tDCS in the gray matter and at three regions of interest in the gray matter, namely, the anodal electrode (FC5), cathodal electrode (FP2), and an independent site remote from the electrodes (PZ). A “canonical HRF” with time and dispersion derivatives and a finite impulse response (FIR) model with three parameters captured the effects of anodal tDCS on the temporal profile of the HRF. The FIR model showed tDCS onset effects on the temporal profile of HRF for verum and sham tDCS conditions that were different from the no tDCS condition, which questions the validity of the sham tDCS (placebo). Here, we postulated that the effects of tDCS onset on the temporal profile of HRF are subserved by the effects on neurovascular coupling. We provide our perspective based on previous work on tES effects on the neurovascular unit, including mechanistic grey-box modeling of the effects of tES on the vasculature that can facilitate model predictive control (MPC). Future studies need to investigate grey-box modeling of online effects of tES on the neurovascular unit, including perivascular space, neurometabolic coupling, and neurovascular coupling, that can facilitate MPC of the tES dose-response to address the momentary (“state”) and phenotypic (“trait”) factors.

## 1. Introduction

Systems modeling (1) of brain responses to transcranial electrical stimulation (tES) can provide a mechanistic understanding of the dose-response, e.g., based on, simultaneous functional near-infrared spectroscopy (fNIRS) and electroencephalogram (EEG) that can elucidate tES dose-response subserved by neurovascular coupling. Neurovascular coupling can be dysfunctional in a pathological state, e.g., post-stroke (2). Here, the transient coupling relationship between changes in the EEG power spectrum and fNIRS hemodynamics signals may capture the effects of tES on the neurovascular unit (NVU) that incorporates vascular smooth muscle, perivascular space, synaptic space, and astrocyte glial cell. We postulated that the online tES effects on the NVU are important (3) to understand the after-effects of tES (4) that can be driven by the perturbation to the state of the neurovascular unit (5). Specifically, the immediate effects of tES are modulation of the state of the brain's extracellular space through the arousal pathway (6), which can modulate the excitation-inhibition (E–I) balance (7). The extracellular space, including the extracellular matrix (8), is crucial to maintain the E–I balance, and the synaptic after-effects of tES can be driven by the E–I homeostatic mechanisms (9) following tES perturbation. Indeed, recent studies are increasingly showing a lack of online modulation of concurrent transcranial magnetic stimulation motor-evoked potentials (MEP) that are generated *via* transynaptic activation of pyramidal cells projected on spinal motoneurons (10). Here, we postulate that the extracellular space, including the extracellular matrix plays a crucial role during online modulation by tES, which is relevant in the optimisation of the tES dose (6). For example, tES modulation of the permeability of the blood–brain barrier (11) can change the substance transport (12) including glucose that can modulate neuronal excitability (13) and neurovascular coupling [for a given insulin action (14, 15)]. Then, the integration of signaling from various tES-evoked sources in the extracellular space can be driven by the E–I homeostatic mechanisms afterwards that may explain the (paradoxical) MEP facilitation during cathodal tDCS at 2.0 mA (16) as well as partially non-linear stimulation intensity-dependent effects (17) (that include neuronal calcium influx effects). Therefore, systems analysis of the online modulation of the neurovascular coupling (3), including the modal analysis (with frequency response function) of the coupling (6), can capture tES dose-response which was not feasible based solely on the MEP (16).

Published animal studies have provided some insights. For example, a study by Han et al. (18) found that the concentration of oxyhaemoglobin increased almost linearly during tDCS and then decreased linearly immediately after the end of tDCS with a similar rate of change that differed from rat to rat. Han et al. (18) suggested that individual differences in anodal tDCS' neuronal after-effects may be related to individual variability in the rate of change of haemodynamic response to tDCS. In their study (18), the concentration of deoxygenated hemoglobin did not show a significant difference during and after tDCS (18). Direct effects of tDCS on cortical astrocytes with astrocyte regulation of blood flow (19) are possible without changes in the local field potential (20) that can also lead to dilation or constriction of the arterioles (21). Wachter et al. (22) showed sustained polarity-specific changes in cerebral blood flow (CBF) using laser Doppler blood perfusion imaging (LDI), where the duration and degree of CBF changes depended on the intensity of the current applied. Furthermore, Mielke et al. (23), using LDI, showed a regionally limited, long-lasting, and reversible decrease in hemispheric CBF due to cathodal tDCS that depended on the current intensity and the size of the stimulation electrode. It is crucial to note that the tDCS-effected haemodynamic changes are not in the large vessels that are probed with arterial spin labeling (ASL) MRI in human studies (24). Here, intravoxel incoherent motion MRI (25) may be more suitable to capture the tES effects.

We showed that the physiological state of the extracellular space, including the perivascular space and metabolites, can be modulated by tES (1). The tES effects on neuronal calcium have been proposed as one of the mechanisms subserving partially non-linear dose-response (17). Notably, tES effects on astrocytes (26) can lead to the release of neuronal NO synthase in the extracellular space that is fundamental to neurovascular coupling (27). Here, tES-led activation of astrocytic calcium signaling (20) can mediate neurovascular signaling to capillary pericytes (28). In addition, there may be an interaction between astrocytes and microglia (29, 30) that can modulate purinergic neurovascular signaling (28, 31). Here, the arousal effects of tES (6) will also be important in modulating neurovascular coupling since norepinephrine controls astroglial responsiveness to local circuit activity (32). Then, extracellular potassium can increase due to an increase in calcium ions in the astrocyte end feet due to tES (20) that we have modeled earlier (1). We (1) have explicitly modeled the inward-rectifying potassium channels in the vascular smooth muscle cells (SMCs) that are sensitive to increases in extracellular potassium. Here (1), the gray-box modeling was based on experimental data from previous works using fNIRS (33) and fNIRS in conjunction with electroencephalography (EEG) (3). Prior works postulated that tES led to an increase in interstitial K^+^ that can modulate the neurovascular system's sensitivity through Kir channels (34) and the interaction with calcium activity in the capillary pericytes (5, 35). Importantly, modulation of the blood-brain barrier by tES (11) can have morphological effects where Minager et al. (36) recently found perivascular space morphological changes in response to tES. Therefore, the perivascular pathway of tES effects *via* astrocytes and vasculature, postulated by Arora et al. (1), needs further investigation. Then, the basal ganglia perivascular space volumes showed greater tES sensitivity than white matter perivascular space volumes which may be related to the different morphologies (37), e.g., basal ganglia perivascular space connected straight to subarachnoid space providing a straight high conductivity tES current path (38). Then, even partial failure of the blood-brain barrier and substance transport can change the osmotic gradients leading to a change in the volume of the perivascular space according to published mathematical modeling (39). In principal accordance to all these aforementioned effects of tES on the NVU, we postulate that a canonical haemodynamic response function (HRF) with univariate model (40) using a single dilation parameter severely limits the physiological interpretability of the expected neurovascular coupling modulation by tES (2, 3, 41). Here, an informed choice of the HRF model (42) is crucial to elucidate the tES effects on the brain, i.e., the dose-response.

The choice of the HRF model can range from canonical HRF that can use a single dilation parameter or two parameters—time and dispersion derivatives—to fit the evoked haemodynamic response, e.g., blood oxygenation level dependent (BOLD), to a more flexible finite impulse response (FIR) model with more parameters, e.g., response height (RH), time to peak (TTP), and full width at half maximum (FWHM), to non-parametric impulse response estimation from linear systems analysis [(43), p. 1]. Here, the challenge is that the model selection can result in bias in addition to a loss in power (42), e.g., model order selection for non-parametric impulse response estimation may need extensive analysis in terms of power, bias, and parameter confusability (42). The goal of this perspective article is to highlight this aspect with well-defined parameters, e.g., time and dispersion derivatives, RH, TTP, and FWHM, that can be investigated as the basis of the HRF temporal profile. The analysis of the HRF temporal profile is important since Ekhtiari et al. (44) published a checklist to evaluate the methodological quality of concurrent studies of functional magnetic resonance imaging (fMRI) studies; however, the protocol did not elaborate on the methods for separating tES effects on the neuronal activation from the effect on the NVU or neurovascular coupling (41), i.e., the HRF. Here, mapping of the tES stimulus-related BOLD signals measured using fMRI is achieved by fitting a general linear model (GLM) to the time course with a pre-specified canonical HRF model, e.g., double-gamma function (40). Then, Vincent et al. (45) proposed spatial localization of HRF that addresses HRF recovery and localization of cerebral activity using a black-box finite impulse response (FIR) and temporally regularized FIR models. Arora et al. (41) published a biophysically informed neurovascular coupling model to capture the haemodynamic response to tES based on functional near-infrared spectroscopy (fNIRS). Arora et al. (41) addressed the challenge to find a trade-off between estimation bias and overfitting to fNIRS data by reducing the degrees of freedom in a grey-box model. Grey-box modeling can also be applied to BOLD data for HRF recovery, e.g., one HRF per voxel, which can be used on either volume-based data sets or on data projected onto the cortical surface to reduce the computational needs for inference (45). Here, the solution with the best fit to the electric field distribution, that is, the source signal, can be selected using a cost function with regularization in the tES-fMRI studies. In this article, we applied an estimation of the HRF model based on well-defined parameters, i.e., time and dispersion derivatives, RH, TTP, and FWHM using an open-source fMRI-tDCS data set (46), to elucidate the effects of tES on the HRF temporal profile as the flexibility of the HRF model increased from two parameters to three parameters. It was postulated that three parameters will allow for tES onset response, the peak, and the post-stimulation undershoot that is expected based on grey-box modeling (1).

## 2. Methods—fMRI-tDCS HRF fitting

Arora et al. (1) presented the minimal realization transfer functions for the four pathways based on the fNIRS-tDCS data set (46) that provided a qualitative analysis of the haemodynamic response. The four pathways from the tES as input to the vessel response as output (1) were:

(a) Pathway 1: Synaptic potassium → vessel circumference.
(b) Pathway 2: Astrocytic current channel → vessel circumference.
(c) Pathway 3: Perivascular potassium → vessel circumference.
(d) Pathway 4: Smooth muscle cell → vessel circumference.

Arora et al. (1) found that the tDCS perturbation Pathway 4 had the fastest response (peaked at 0.4 s) and the tDCS perturbation Pathway 1 had the slowest response (peaked at 5 s). In this perspective article, we used an open-source tES-fMRI data set (46) that showed no field inhomogeneity using functional sensitivity metrics in the gray matter during 2 mA anodal tDCS that was delivered for 20 min to the frontal cortex (anode at FC5 and cathode at FP2 in a 10–20 system). The fMRI data were also collected during sham-tDCS and no-tDCS conditions which allowed us to compare the tDCS onset response [discussed in detail by Arora et al. (1)]. Here, sham-tDCS is postulated to evoke an onset response [discussed in detail by Arora et al. (1)]; however, the no-tDCS condition should not have any onset response. We used “canonical HRF” with time and dispersion derivatives and an “FIR HRF” model with RH, TTP, and FWHM using the rsHRF toolbox (47) to elucidate the effects of differences in the tDCS onset on the temporal profile of the HRF. The FIR model was found using the “rsHRF_estimation_FIR” function in the rsHRF toolbox (47), while the canonical HRF model was found using the “rsHRF_estimation_temporal_basis” function in rsHRF toolbox. Here, the HRF estimation elucidated the effects of anodal tDCS on the HRF temporal profile in the gray matter and at the three regions of interest (ROIs) in the gray matter underlying anodal electrode (FC5), cathodal electrode (FP2), and an independent site remote from the electrodes (PZ).

## 3. Results: fMRI-tDCS HRF fitting

**Figure 1:**
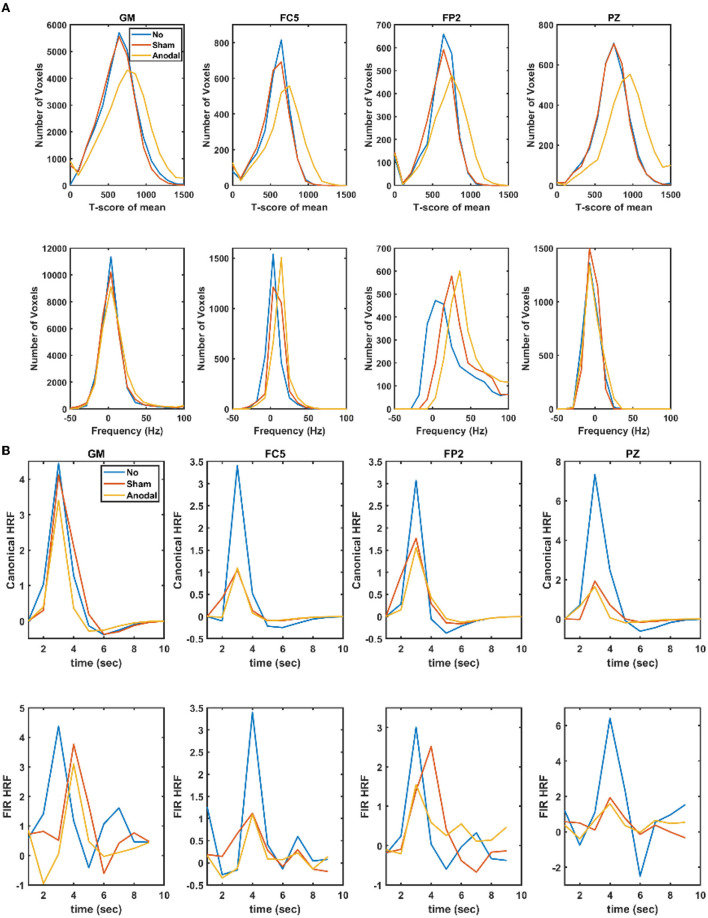
**(A)** Functional sensitivity metrics: the top row shows the *t*-score of the mean in the gray matter (GM) and gray matter ROIs, FC5 (anodal electrode), FP2 (cathodal electrode), and PZ (remote location), while the bottom row shows the corresponding offset frequency (in Hz) to capture field inhomogeneity. **(B)** Estimated haemodynamic response function (HRF) in the gray matter (GM) and the gray matter ROIs, FC5 (anodal electrode), FP2 (cathodal electrode), and PZ (remote location) where the top row shows the results using canonical HRF with time and dispersion derivatives, while the bottom row shows the results using a finite impulse response model (FIR). No, no-tDCS condition; Sham, sham-tDCS condition; Anodal, anodal-tDCS condition.

**Figure 2:**
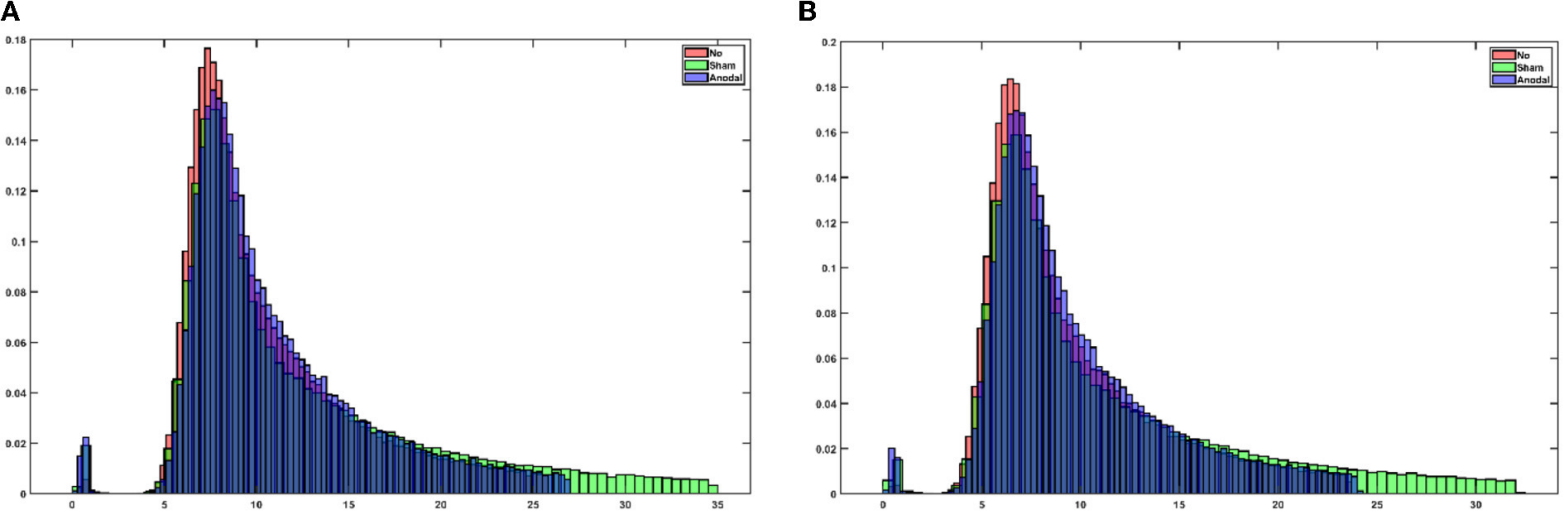
**(A)** An estimation of the probability density function across all voxels for the height parameter (in the *x*-axis) of the HRF using canonical HRF with time and dispersion derivatives. **(B)** Estimation of the probability density function across all the voxels for the height parameter (in the *x*-axis) of the HRF using the finite impulse response (FIR) model.

Supplementary Figure S1 shows the electrical field distribution in the gray matter for 2 mA tDCS with FC5 (anodal electrode) and FP2 (cathodal electrode) computed with the ROAST package (48) including tES evoked blood volume effects on the brain tissue conductivity. Figure 1A shows the functional sensitivity metrics calculated using the open-source code and data from the tES-fMRI data set (46), where anodal tDCS led to a shift to a higher *t*-score (first row of Figure 1A) as expected (49). At the same time, the width of the frequency distributions (second row of Figure 1A) remained similar across conditions, reflecting similar field inhomogeneity. Then, we applied HRF estimation using the rsHRF toolbox (47) that elucidated the effects of anodal tDCS at four ROIs in the gray matter based on “canonical HRF” with time and dispersion derivatives and an “FIR HRF” model with RH, TTP, and FWHM. Figure 1B shows that the canonical HRF with time and dispersion derivatives mainly captured the tES effects on the magnitude of the main response and the magnitude of the undershoot. In contrast, the FIR model better captured the impact on the whole temporal profile of the HRF due to the flexible HRF model with three parameters. Here, we observed that both the anodal tDCS condition and the sham tDCS condition had similar FIR at the FC5 (anodal electrode) and PZ (remote location) ROIs that captured the onset response to tDCS in both conditions; however, those were found to be different from the non-tDCS condition, i.e., the proposed tES onset response is present in the case of sham tDCS (1) but tES onset response is missing when no tDCS perturbation is applied as expected. For the ROI FP2 (cathodal electrode), the FIR-based HRFs differed between all conditions, specifically anodal tDCS and sham tDCS conditions. Figure 2 shows an estimation of the probability density function across all voxels for the height parameter of the HRF found for both the “canonical HRF” with time and dispersion derivatives and an “FIR HRF” model using the open-source rsHRF toolbox (47). In Figure 2, the no-tDCS condition (shown with red color bars) led to a higher expectation (based on probability density) of a lower height parameter than the sham-tDCS (green color) and the anodal-tDCS (blue color) conditions from both the canonical HRF with time and dispersion derivatives and FIR HRF models. Here, the tail of the sham-tDCS (shown with green color bars) shifted the expectation toward a higher HRF height parameter (on the *x*-axis) when compared with that of the anodal-tDCS (shown with blue color bars) conditions. The estimation of the HRF height parameter was not found to be different between the “canonical HRF” with time and dispersion derivatives and the “FIR HRF” model. However, when we investigated the HRF temporal profile, as shown in Figure 1, the “FIR HRF” model captured the onset response (“initial dip” in the bottom panel of Figure 1B), which was not captured by the “canonical HRF” with time and dispersion derivatives (top panel of Figure 1B). In the case of the “canonical HRF” with time and dispersion derivatives, the no-tDCS and sham-tDCS conditions resulted in a similar temporal profile that was different from the anodal tDCS condition. This clearly demonstrated the advantage of greater flexibility in the “FIR HRF” model due to its three parameters when compared to the “canonical HRF” with only two parameters—time and dispersion derivatives.

## 4. Perspective on the effects of transcranial electrical stimulation—at onset and longer term

We analyzed an open-source fMRI-tDCS data set (46) that had a TR = 3.36 s, thus, it could capture 0.052–0.145 Hz activity associated with smooth muscle cell activity (50). Our study found based on the fMRI-tDCS data set (46) that both the anodal tDCS condition and sham tDCS condition had similar FIR HRF model at the FC5 (anodal electrode) and PZ (remote location) ROIs. Then, for the FP2 (cathodal electrode) ROI, the FIR-based HRFs were different across all conditions, specifically, anodal-tDCS and sham-tDCS conditions, which may be related to the modulation of local cortical inhibitory circuits and its interaction with the stimulation of the perivascular nerves and astrocytes [discussed in Arora et al. (1)]. Arora et al. (1) proposed, based on computational analysis, that the onset of tDCS can directly stimulate the perivascular nerves and can directly affect astrocytes, as highly conductive cerebrospinal fluid provides a highly conductive route to current flow (51) which was also shown based on computational modeling (38). Khadka and Bikson (38) also suggested that blood-brain barrier polarization may lead to direct modulation of blood-brain permeability (11) superseding direct tES neuronal regulation (52), especially at higher current intensity (53). In fact, our recent results showed perivascular space morphological changes in response to tES (36) which questions the safety of higher 4 mA stimulation (53). Furthermore, the permeability of the blood-brain barrier can modulate neuronal function since blood contains molecules, for example, glucose and ligands for neural and glial receptors (52) where the spatiotemporal dynamics of the tES may be important in neuronal and extracellular state modulation. Various tES modalities have differences in the temporal profile of current stimulation. In tDCS, the current profile has a monophasic, non-oscillating constant value, and steady-state blood-brain barrier permeability changes during longer duration cathodal tDCS at higher 2.0 mA (and not at lower 1.0 mA) may explain the (paradoxical) facilitation of MEP (16), e.g., due to the glucose and albumin leakage to parenchyma (54). In contrast, in transcranial alternating current stimulation (tACS), the oscillating current reverses the flow rhythmically at a specific frequency. That is, tACS differs from tDCS in that it provides a mechanism for manipulating intrinsic oscillations through the injection of sinusoidal currents that can be therapeutic (55). The other methods are transcranial oscillating current stimulation (tOCS) that uses tDCS to set a baseline to the tACS oscillations and transcranial random noise stimulation (tRNS) that injects “noisy” current with bounded stochasticity. Because tES modulatory effects on blood vessels can be mediated by multiple pathways in the NVU, a deeper understanding of the signaling pathways will be crucial for a mechanistic understanding of tES effects, including its entrainment effects on neurons and blood vessels (50), where the onset response to tES oscillatory peaks may even be optimized for therapy (56).

The onset response in the case of a short-duration sham tES is also important, as found in the current study, which may explain the hidden source of variability in the tES after-effects (57). Significantly, short-duration (<3 min) tDCS can have physiological effects in terms of the response to tES onset, which has been discussed in prior studies (41, 58), where biological effects can extend beyond intended transient sensations (57). For example, sensory stimulation can also evoke arousal (6) with brain response (59) that can be relevant in acute stroke therapy (60) and cognitive impairment (61). However, sensory stimulation can have both beneficial and detrimental effects, viz., the potential risk from increased metabolic demand (60), where a mechanistic understanding will require physiologically detailed modeling, invasive animal studies, and systems biology approaches (41). We postulate that short-duration tES can act through superficial nerves (62), noradrenergic axons in the brain (29), and efferent innervation (63). The autonomic nervous system's efferent innervation is known to regulate metabolic organs including systemic glucose uptake *via* insulin independent mechanism (64) and neurohormonal stress axes (65). Here, a marker is tonic pupil dilation mediated by sympathetic output acting against parasympathetically mediated pupil constriction (66). Sympathetic nerves increase blood nutrient factors such as glucose by promoting gluconeogenesis, which involves the inhibition of insulin secretion, while parasympathetic nerves inhibit gluconeogenesis that indirectly involves the promotion of insulin secretion (67). Therefore, mechanistic understanding of the interaction of changes in glucose concentration with the neurovascular tissue function during tES is relevant for its safe delivery in acute stroke therapy (60). In this study, sympathoexcitation (68) is postulated to affect the smooth muscle cells where the related oscillatory frequencies (0.021–0.145 Hz) are primarily in the pial, penetrating, and precapillary arterioles. Capillaries have a lower oscillatory frequency of 0.01–0.02 Hz that is postulated to reflect the Fahraeus–Lindqvist effect (69), that is, the non-linear dependence of apparent blood viscosity on haematocrit and vessel diameter that can also determine the scaling of the background power law at rest and under task (70). Blood viscosity and blood glucose have a direct relationship, e.g., cognitive load and long-duration tDCS (71) can lead to a significant reduction in blood glucose. The amount of cognitive load associated with task performance is an index of its sensitivity to enhancement by glucose (72) that can affect the lower oscillatory frequency of 0.01–0.02 Hz, as well as the background power-law scaling (70). Investigation of tDCS effects on the neurometabolic state will require augmentation of the NVU model (1) with metabolic pathways from Jolivet et al. (73), as discussed next.

Stoichiometry (glycolysis and glycogenolysis pathway) will determine oxygen to glucose consumption (OGI = CMR 2/CMRlc) (74). A reduction in OGI (~6) (75) can lead to the accumulation of lactate [(76) modeled by (73)] without an associated reduction in oxygen supply [(77); glycolysis over respiration]. Jolivet et al.'s model (73) is divided into four main compartments, namely, a neuronal compartment, an astrocytic compartment, the extracellular space, and the vascular compartment where the extracellular space will be mapped to synaptic space—refer to Arora et al. (1). It can be assumed that the electric field can linearly modify the average membrane potential (*V*) of the different neuronal subpopulations [within a certain range of intensity; (78)], i.e., Δ*V* = λ.*E*, where the electric field (*E*) can be modeled as an anatomically realistic full-head model. Membrane polarization will lead to synaptic after-effects that will lead to vessel response as presented in our prior study (41). Therefore, a neuronal compartment needs to be added to our neurovascular coupling model (41) containing voltage- and calcium-gated ion channels following the Hodgkin-Huxley formalism from Jolivet et al.'s model (73). Based on Jolivet et al.'s model (73), neurons can be divided between a cytosolic subcompartment and a mitochondrial sub-compartment to account for the compartmentalisation of oxidative and glycolytic metabolisms. In this study, a tDCS-applied electric field can linearly modify the average membrane potential (*V*) of the different neuronal subpopulations (78) to change the E–I ratio (7) and OGI (73). Then, it is assumed that the current density at the scalp (*I*_*tdcs*_) is proportional to the current density (*J*_*tDCS*_) in the neurovascular brain tissue based on a lead field matrix leading to the vasoactive signal *via* a first-order transfer function:


(1) $$\begin{array}{l} v_{i}=\;\frac{\lambda }{s/\tau +1}I_{tdcs} \end{array}$$

where λ is arbitrary gain from the lead field matrix and τ is the time constant (41). The grey-box modeling in our prior work (1) was for short-duration tDCS effects, which need to be augmented with metabolic signaling (79) to capture longer-duration tES effects.

### 4.1. Pathway 1: tDCS perturbation of synaptic potassium leading to changes in vessel circumference

Studies have shown that potassium (K^+^) can act as a potent vasodilator signal (80, 81) that couple local neuronal activity to vasodilation in the brain and have a significant role in cerebrovascular mechanisms. Studies have shown that the potassium pathway is responsible for the fast onset of vasodilation compared to the other mediators. In Pathway 1, the potassium concentration in the synaptic space is modulated by the local tDCS current. In this study, *J*_*K*_*s*__ is the K^+^ released from active neurons that are considered to be perturbed by tDCS from its baseline condition such that $J_{K_{s}}=v_{1}=\;\frac{K_{1}}{s/\tau +1}I_{tdcs}\;$using Equation (1). The potassium concentration in the synaptic space, ${\left[K^{+}\right]}_{s},$ is then given as:


(2) $$\begin{array}{l} \frac{d{\left[K^{+}\right]}_{s}}{dt}=\;J_{K_{s}}+\frac{K_{1}}{s/\tau +1}I_{tdcs}\\ \;-\;J_{\Sigma Kmax}k_{Na}\frac{{\left[K^{+}\right]}_{s}}{{\left[K^{+}\right]}_{s}+\;KKO_{a}} \end{array}$$

where *J*_*K*_*s*__ is the baseline flux of K^+^ in the synaptic space, *J*_Σ*Kmax*_ is the maximum flux, *k*_*Na*_ is a constant parameter that depends on the extracellular sodium concentration, *KKO*_*a*_ is the threshold value for K^+^ concentration in the synaptic space, ${\left[K^{+}\right]}_{s}$. This pathway can be augmented with metabolic signaling (79) based on Jolivet et al.'s equations (73) in the extracellular space that also capture glucose and lactate interactions between the neuron and the astrocyte.

#### 4.2. Pathway 2: Perturbation of the astrocytic transmembrane current by tDCS leading to changes in vessel circumference

Astrocytes are susceptible to small variations in their membrane potential, and their long processes are sensitive to polarization by tDCS (32). Therefore, tDCS can also affect potassium buffering *via* astrocytes (32, 82). In this study, the astrocytic transmembrane current (*I*_*T*_) was perturbed by tDCS, $I_{T}=v_{2}=\;\frac{K_{2}}{s/\tau +1}I_{tdcs}\;$from Equation (1) that was added to other transmembrane currents, including *I*_*BK*_ is current through big potassium (BK) channel, *I*_*leak*_is leak current, *I*_*TRPV*_is electrical current through the TRPV channel, and *I*_Σ*K*_ is the electrical current carried by the K^+^ influx at the perisynaptic process:


(3) $$\begin{array}{l} \frac{dV_{k}}{dt}=\;\frac{1}{C_{astr}}\\ \;\left(I_{BK}-I_{leak}-\;I_{TRPV}-\;I_{\Sigma K}+\frac{K_{2}}{s/\tau +1}I_{tdcs}\;\right) \end{array}$$

A mitochondrial sub-compartment can be added from Jolivet et al.'s study (73) to account for the compartmentalisation of oxidative and glycolytic metabolisms. In this study, astrocytes are known to contain a metabolic network including glycolytic enzymes, lactate dehydrogenase, glucose and lactate transporters, NADH shuttles, oxidative metabolism, phosphocreatine, and the Na, K-ATPase electrogenic pump.

### 4.3. Pathway 3: Perturbation of perivascular potassium concentration by tDCS leading to changes in vessel circumference

Glial cells maintain extracellular K^+^ concentration by the imbalance in their membrane polarity and can affect K^+^ spatial buffering, affecting tDCS modulation (32, 82). Astrocytic release of K^+^, *via* two potassium channels (BK and KIR), into the perivascular space can be perturbed by tDCS. Astrocytic role in neurovascular coupling may be related to the strength of stimulation where high strength can lead to vasoconstriction [(83); mediated *via* K^+^ and EET signaling (80)]. Vasoconstriction can follow vasodilation when the astrocytic calcium concentration (or perivascular K^+^ concentration) increases above a certain threshold. We assumed that low-intensity tDCS perturbation would not cross that threshold where ${\left[K^{+}\right]}_{T}=v_{3}=\;\frac{K_{3}}{s/\tau +1}I_{tdcs}$. Thus, the perivascular potassium concentration, ${\left[K^{+}\right]}_{P}$, is given as:


(4) $$\begin{array}{l} \frac{d{\left[K^{+}\right]}_{P}}{dt}=\;\frac{J_{BK\;}}{VR_{pa\;}}+\;\frac{J_{KIR\;}}{VR_{ps\;}}-\;R_{decay}\left({\left[K^{+}\right]}_{P}-\;{\left[K^{+}\right]}_{P,min}\right)\\ \;+\frac{K_{3}}{s/\tau +\;1}I_{tdcs.} \end{array}$$

In this study, ${\left[K^{+}\right]}_{P,min}$ is the resting-state equilibrium K^+^ concentration in the perivascular space. The K^+^ flow from the astrocyte and SMCs is *J*_*BK*_ and *J*_*KIR*_, corresponding to BK and inward rectifying potassium (KIR), respectively. *VR*_*pa*_ and *VR*_*ps*_ are the volume ratios of perivascular space to astrocyte and SMC, respectively. *R*_*decay*_ is the rate at which perivascular K^+^ concentration decays to its baseline state.

### 4.4. Pathway 4: tDCS perturbation of the voltage-gated ion channel current on the smooth muscle cell leading to changes in vessel circumference

The potassium channels (KV) of SMCs and inwardly rectifying K^+^ channels are important in penetrating arterioles that control arterial diameter by exerting a major hyperpolarising influence (84). Therefore, the local tDCS electric field (38) can perturb the voltage-gated potassium current ($\Delta \;I_{KV}=v_{4}=\;\frac{K_{4}}{s/\tau +1}I_{tdcs}$) that was added to other currents including *I*_L_, *I*_K_, *I*_Ca_, and *I*_KIR_ that represent leak, K^+^, Ca^2+^, and KIR channel currents, respectively, in the SMC compartment. Then, the SMC membrane potential, *V*_SMC_, is given by:


(5) $$\begin{array}{l} \frac{dV_{SMC}}{dt}=\;\frac{1}{C_{SMC}}\\ \;\left(-I_{L}-\;I_{K}-I_{Ca}-I_{KIR}-\;I_{KV}+\frac{K_{4}}{s/\tau +1}I_{tdcs}\;\right). \end{array}$$

In this study, the four tDCS perturbation pathways are nested (1), that is, Pathway 1 is represented by 17 ordinary differential equations starting from synaptic K^+^ that nested other tDCS perturbation Pathways 2–4, which results in vessel oscillations that critically depend on the parameters, including vessel stiffness, in the ordinary differential equations.

The therapeutic effects of tES on brain metabolism are relevant in dementia (61) since the early stage of Alzheimer's disease (AD) shares molecular and cellular features, including insulin resistance and mitochondrial dysfunctions (85), where AD has been called “type 3 diabetes” (86–88). Elevated glucose levels during long-standing diabetes have been shown to induce structural and functional changes in different proteins in the body, including albumin, globulins, fibrinogen, and collagens (89). Both the replication of protein aggregates and their spreading throughout the brain are implicated in the progression of AD (90). Also, cross-linking of proteins by advanced glycation end products in the vessel wall increases vascular stiffness, and modification of extracellular matrix proteins decreases pericyte adherence which can lead to neurovascular uncoupling (61) and reduced oxygen supply in the brain (91). Then, accelerated cognitive decline is postulated because of changes in small vessel structure and function, specifically expansion of the basement membrane and a loss of vascular cells (92). However, diabetes can lead to both microvascular and macrovascular complications (92). Rouch et al. (93) showed that mainly arterial stiffness is associated with mild cognitive impairment (MCI) at a higher risk of dementia. The study on 375 elderly ambulatory subjects with MCI showed that only an increased arterial stiffness was associated with the conversion of MCI to dementia, whereas intima-media thickness, carotid plaques, or carotid artery diameter were not after controlling for age and other confounding factors. In this study, vessel stiffness is a crucial factor in determining oscillatory peaks of hemodynamics, estimated with modal analysis (6), and can be probed in health and disease using responses evoked by tACS of total hemoglobin (blood volume), as demonstrated for human-in-the-loop optimisation (6). Then, human-in-the-loop optimisation of tES can also address momentary (“state”) and phenotypic (“trait”) factors (94). These vessel oscillations are known to be important not only for supporting higher oxygen availability distant from small vessels (69) but also for supporting waste clearance within the brain parenchyma, specifically convective bulk flow drainage along the basement membrane of capillaries and arterial walls (95). Therefore, changes in blood vessel pulsatility, including expansion of the basement membrane (92), can alter convective bulk flow drainage that is important to prevent accumulation of concerning neurotoxic waste protein, e.g., protein aggregates. In a large cohort study (96), younger age at onset of diabetes was significantly associated with a higher risk of subsequent dementia. Interestingly, reduced glucose availability in the CNS can also directly trigger behavioral deficits by promoting the development of amyloid beta and tau neuropathology as well as synaptic dysfunction. In this study, reduced glucose availability in the CNS can be related to small vessel dysfunction, which also reduces oxygen availability away from the small vessels (69).

**Figure 3:**
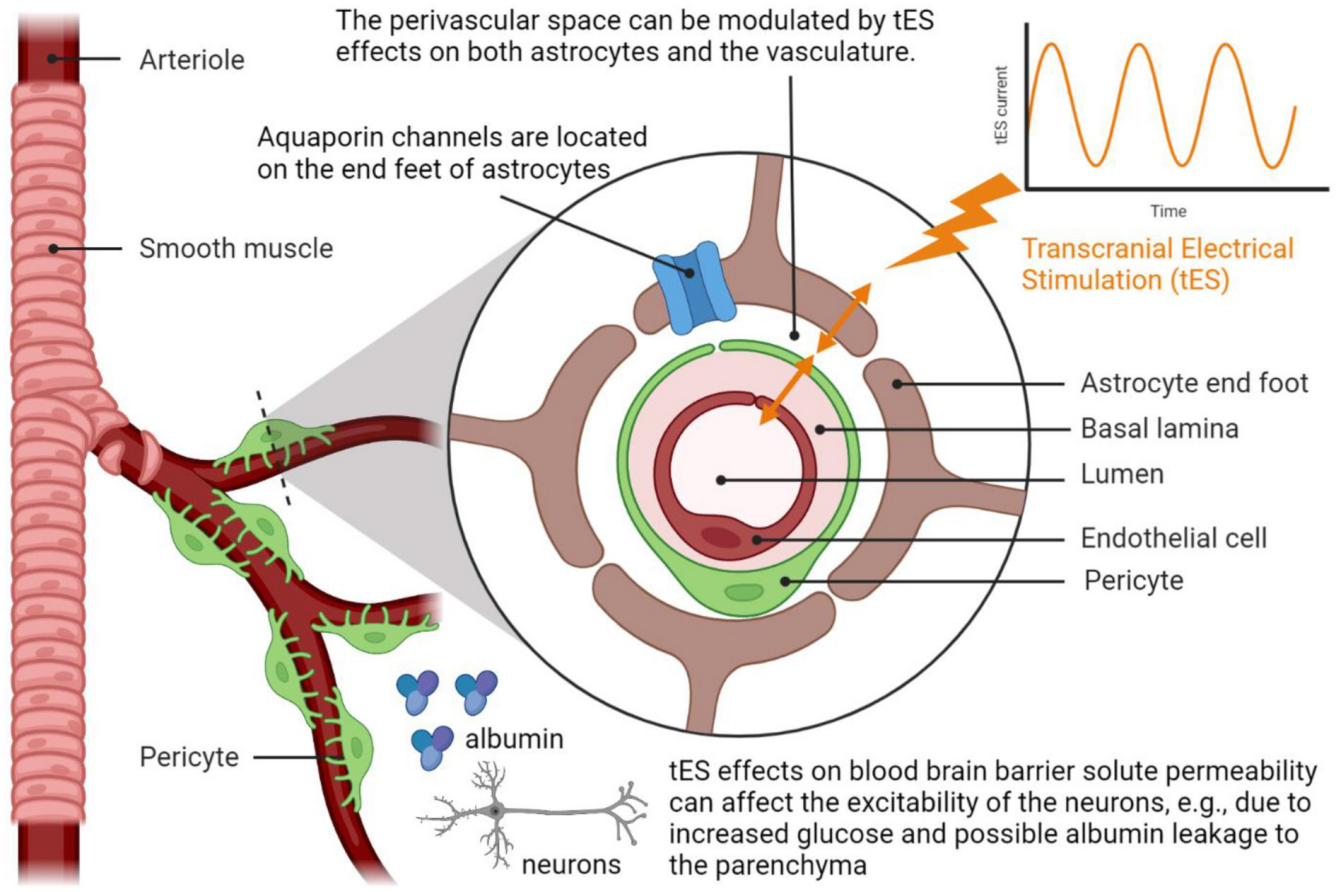
Transcranial electrical stimulation can acutely modulate perivascular space morphology by affecting the interactions between the astrocyte end feet that cover smooth muscle cells and endothelial cell walls, i.e., the perivascular pathway elucidated by Arora et al. (1). Neuroimaging evidence of the modulation of the perivascular space morphology from Minager et al. (36). Created with BioRender.com.

Figure 3 shows the postulated mechanism for tES modulation of the perivascular space based on the tES effects on both astrocytes and the vasculature (1). In this study, Arora et al. (1) postulated tES modulation of the vasculature *via* the perivascular pathway, where vasoconstriction can increase while vasodilation can decrease the volume of the perivascular space (refer to Figure 3). In the computational model (1), an immediate vascular response was captured through the perivascular pathway by the interaction between the perivascular potassium and calcium concentration that led to steady-state stand-alone steady-state vessel oscillations <0.1 Hz. These vessel oscillations can be entrained by neuronal oscillations (97) due to the shared extracellular space in the NVU, and neurovascular coupling can be investigated using joint imaging with fNIRS-EEG (3, 54). Then, the fast vascular response at the onset of tES (1) can modulate the perivascular flow, where human-in-the-loop optimisation may be feasible based on the blood volume (total hemoglobin) feedback from fNIRS (6). In our prior study (6), we found from modal analysis and a case study in a healthy human that the “optimal” oscillatory frequency was close to 1 Hz, meriting mechanistic investigation with respect to astrocytes and interstitial potassium. In this study, short-term (<150 s) acute tES can affect the vasculature (41) for immediate control of blood vessel response using model predictive control (MPC) (6). MPC uses an internal model of the interaction of cortical activity, local metabolic factors, and the vascular response to make predictions of the system behavior, considering neurovascular dynamics over a predefined prediction horizon, to optimize tES control actions. For online operation, MPC operates in a receding horizon fashion, i.e., new system measurements and new predictions into the future are made at each time step. It is postulated that an optimal tES oscillatory pattern can be therapeutically beneficial for acute effects on the vasculature and/or astrocyte end feet, e.g., to modulate Fahræus-Lindqvist-driven oscillations (98) as well as to modulate perivascular volume (and fluid movement; refer to Figure 3). Acute modulation of perivascular volume with tES is supported by Minager et al. (36) that showed tES modulation of perivascular space morphology which may also be related to the vascular response to tDCS (1, 50). Since the portable neuroimaging approach is amenable to a point-of-care setting (6) when compared to the MRI-tES (36, 46), portable neuroimaging based MPC of tES during sleep may even facilitate the glymphatic clearance (99). Here, the tES pattern can be optimized at the point-of-care setting (6) to evoke the necessary blood volume response (output) to limit potential risk from an increased metabolic demand (60) in pathological tissue (e.g., ischaemic in vascular dementia). We postulate that the therapeutic application of tES may transition the NVU (2, 3, 100) to an improved neurovascular system sensitivity (5) for beneficial cognitive after-effects, e.g., may reduce cognitive fatigue (101) due to enhanced extracellular clearance.

The limitation of the current study includes the unavailability of the fMRI-EEG data to capture the long-term effects of tES on neurovascular coupling. Our fNIRS-EEG data has been shown to be feasible for computational modeling of neurovascular coupling (3, 41). Also, the trade-off between bias (in canonical HRF) and variance (in FIR HRF) achieved by applying mechanistic gray-box modeling of the NVU pathways to fMRI-tES data was not demonstrated in this perspective article which is undergoing analysis for our future research article.

## Data availability statement

The original contributions presented in the study are included in the article/Supplementary material, further inquiries can be directed to the corresponding author.

## Author contributions

YA: data curation, formal analysis, investigation, methodology, software, validation, visualization, and writing—original draft. AD: conceptualization, formal analysis, investigation, methodology, project administration, resources, supervision, validation, visualization, and writing—original draft and review and editing. Both authors contributed to the article and approved the submitted version.

## References

[1] Arora Y Walia P Hayashibe M Muthalib M Chowdhury SR Perrey S. Grey-box modeling and hypothesis testing of functional near-infrared spectroscopy-based cerebrovascular reactivity to anodal high-definition tDCS in healthy humans. PLoS Comput Biol. (2021) 17:e1009386. 10.1371/journal.pcbi.1009386 34613970 PMC8494321

[2] Dutta A Jacob A Chowdhury SR Das A Nitsche MA. EEG-NIRS based assessment of neurovascular coupling during anodal transcranial direct current stimulation–a stroke case series. J Med Syst. (2015) 39:205. 10.1007/s10916-015-0205-7 25686912

[3] Sood M Besson P Muthalib M Jindal U Perrey S Dutta A. NIRS-EEG joint imaging during transcranial direct current stimulation: online parameter estimation with an autoregressive model. J Neurosci Methods. (2016) 274:71–80. 10.1016/j.jneumeth.2016.09.008 27693293

[4] Dutta A. Bidirectional interactions between neuronal and hemodynamic responses to transcranial direct current stimulation (tDCS): challenges for brain-state dependent tDCS. Front Syst Neurosci. (2015) 9:107. 10.3389/fnsys.2015.00107 26321925 PMC4530593

[5] Dutta A. Simultaneous functional near-infrared spectroscopy (fNIRS) and electroencephalogram (EEG) to elucidate neurovascular modulation by transcranial electrical stimulation (tES). Brain Stimul. (2021) 14:1093–4. 10.1016/j.brs.2021.07.019 34333167

[6] Arora Y Dutta A. Human-in-the-loop optimization of transcranial electrical stimulation at the point of care: a computational perspective. Brain Sci. (2022) 12:1294. 10.3390/brainsci12101294 36291228 PMC9599464

[7] Dagar S Chowdhury SR Bapi RS Dutta A Roy D. Near-infrared spectroscopy – electroencephalography-based brain-state-dependent electrotherapy: a computational approach based on excitation–inhibition balance hypothesis. Front Neurol. (2016) 7:123. 10.3389/fneur.2016.00123 27551273 PMC4976097

[8] Dzyubenko E Fleischer M Manrique-Castano D Borbor M Kleinschnitz C Faissner A. Inhibitory control in neuronal networks relies on the extracellular matrix integrity. Cell Mol Life Sci. (2021) 78:5647. 10.1007/s00018-021-03861-3 34128077 PMC8257544

[9] Pozo K Goda Y. Unraveling mechanisms of homeostatic synaptic plasticity. Neuron. (2010) 66:337–51. 10.1016/j.neuron.2010.04.028 20471348 PMC3021747

[10] Klomjai W Katz R Lackmy-Vallée A. Basic principles of transcranial magnetic stimulation (TMS) and repetitive TMS (rTMS). Ann Phys Rehabil Med. (2015) 58:208–13. 10.1016/j.rehab.2015.05.005 26319963

[11] Shin DW Fan J Luu E Khalid W Xia Y Khadka N. *In vivo* modulation of the blood-brain barrier permeability by transcranial direct current stimulation (tDCS). Ann Biomed Eng. (2020) 48:1256–70. 10.1007/s10439-020-02447-7 31916126 PMC7096245

[12] Xia Y Khalid W Yin Z Huang G Bikson M Fu BM. Modulation of solute diffusivity in brain tissue as a novel mechanism of transcranial direct current stimulation (tDCS). Sci Rep. (2020) 10:18488. 10.1038/s41598-020-75460-4 33116214 PMC7595173

[13] Shao L-R Rho JM Stafstrom CE. Glycolytic inhibition: a novel approach toward controlling neuronal excitability and seizures. Epilepsia Open. (2018) 3:191–7. 10.1002/epi4.12251 30564778 PMC6293058

[14] Fernandez AM Martinez-Rachadell L Navarrete M Pose-Utrilla J Davila JC Pignatelli J. Insulin regulates neurovascular coupling through astrocytes. Proc Nat Acad Sci. (2022) 119:e2204527119. 10.1073/pnas.2204527119 35858325 PMC9304019

[15] Zhu WM Neuhaus A Beard DJ Sutherland BA DeLuca GC. Neurovascular coupling mechanisms in health and neurovascular uncoupling in Alzheimer's disease. Brain. (2022) 145:2276–92. 10.1093/brain/awac174 35551356 PMC9337814

[16] Pillen S Knodel N Hermle D Hanke M Ziemann U Bergmann TO. No robust online effects of transcranial direct current stimulation on corticospinal excitability. Brain Stimul. (2022) 15:1254–68. 10.1016/j.brs.2022.08.024 36084908

[17] Batsikadze G Moliadze V Paulus W Kuo M-F Nitsche MA. Partially non-linear stimulation intensity-dependent effects of direct current stimulation on motor cortex excitability in humans. J Physiol. (2013) 591:1987–2000. 10.1113/jphysiol.2012.249730 23339180 PMC3624864

[18] Han C-H Song H Kang Y-G Kim B-M Im C-H. Hemodynamic responses in rat brain during transcranial direct current stimulation: a functional near-infrared spectroscopy study. Biomed Opt Express. (2014) 5:1812–21. 10.1364/BOE.5.001812 24940542 PMC4052913

[19] MacVicar BA Newman EA. Astrocyte regulation of blood flow in the brain. Cold Spring Harb Perspect Biol. (2015) 7. 10.1101/cshperspect.a020388 25818565 PMC4448617

[20] Monai H Ohkura M Tanaka M Oe Y Konno A Hirai H. Calcium imaging reveals glial involvement in transcranial direct current stimulation-induced plasticity in mouse brain. Nat Commun. (2016) 7:11100. 10.1038/ncomms11100 27000523 PMC4804173

[21] Petzold GC Murthy VN. Role of astrocytes in neurovascular coupling. Neuron. (2011) 71:782–97. 10.1016/j.neuron.2011.08.009 21903073

[22] Wachter D Wrede A Schulz-Schaeffer W Taghizadeh-Waghefi A Nitsche MA Kutschenko A. Transcranial direct current stimulation induces polarity-specific changes of cortical blood perfusion in the rat. Exp Neurol. (2011) 227:322–7. 10.1016/j.expneurol.2010.12.005 21147105

[23] Mielke D Wrede A Schulz-Schaeffer W Taghizadeh-Waghefi A Nitsche MA Rohde V. Cathodal transcranial direct current stimulation induces regional, long-lasting reductions of cortical blood flow in rats. Neurol Res. (2013) 35:1029–37. 10.1179/1743132813Y.0000000248 23899548

[24] Liu ML Karabanov AN Piek M Petersen ET Thielscher A Siebner HR. Short periods of bipolar anodal TDCS induce no instantaneous dose-dependent increase in cerebral blood flow in the targeted human motor cortex. Sci Rep. (2022) 12:9580. 10.1038/s41598-022-13091-7 35688875 PMC9187751

[25] Szubert-Franczak AE Naduk-Ostrowska M Pasicz K Podgórska J Skrzyński W Cieszanowski A. Intravoxel incoherent motion magnetic resonance imaging: basic principles and clinical applications. Pol J Radiol. (2020) 85:e624–35. 10.5114/pjr.2020.101476 33376564 PMC7757509

[26] Li N Sul J-Y Haydon PG. A Calcium-induced calcium influx factor, nitric oxide, modulates the refilling of calcium stores in astrocytes. J Neurosci. (2003) 23:10302–10. 10.1523/JNEUROSCI.23-32-10302.2003 14614089 PMC6741013

[27] Hoiland RL Caldwell HG Howe CA Nowak-Flück D Stacey BS Bailey DM. Nitric oxide is fundamental to neurovascular coupling in humans. J Physiol. (2020) 598:4927–39. 10.1113/JP280162 32785972

[28] Mishra A Reynolds JP Chen Y Gourine AV Rusakov DA Attwell D. Astrocytes mediate neurovascular signaling to capillary pericytes but not to arterioles. Nat Neurosci. (2016) 19:1619–27. 10.1038/nn.4428 27775719 PMC5131849

[29] Mishima T Nagai T Yahagi K Akther S Oe Y Monai H. Transcranial direct current stimulation (tDCS) induces adrenergic receptor-dependent microglial morphological changes in mice. eNeuro. (2019) 6:ENEURO.0204-19.2019. 10.1523/ENEURO.0204-19.2019 31444225 PMC6751370

[30] Gellner A-K Reis J Fiebich BL Fritsch B. Electrified microglia: impact of direct current stimulation on diverse properties of the most versatile brain cell. Brain Stimul. (2021) 14:1248–58. 10.1016/j.brs.2021.08.007 34411753

[31] Bisht K Okojie KA Sharma K Lentferink DH Sun Y-Y Chen H-R. Capillary-associated microglia regulate vascular structure and function through PANX1-P2RY12 coupling in mice. Nat Commun. (2021) 12:5289. 10.1038/s41467-021-25590-8 34489419 PMC8421455

[32] Monai H Hirase H. Astrocytic calcium activation in a mouse model of tDCS—extended discussion. Neurogenesis. (2016) 3. 10.1080/23262133.2016.1240055 27830161 PMC5079391

[33] Muthalib M Besson P Rothwell J Ward T Perrey S. Effects of anodal high-definition transcranial direct current stimulation on bilateral sensorimotor cortex activation during sequential finger movements: an fNIRS study. Adv Exp Med Biol. (2016) 876:351–9. 10.1007/978-1-4939-3023-4_44 26782232

[34] Moshkforoush A Ashenagar B Harraz OF Dabertrand F Longden TA Nelson MT. The capillary Kir channel as sensor and amplifier of neuronal signals: modeling insights on K+-mediated neurovascular communication. Proc Natl Acad Sci USA. (2020) 117:16626–37. 10.1073/pnas.2000151117 32601236 PMC7368319

[35] Glück C Ferrari KD Binini N Keller A Saab AS Stobart JL. Distinct signatures of calcium activity in brain mural cells. Elife. (2021) 10:e70591. 10.7554/eLife.70591 34227466 PMC8294852

[36] Minagar A Sepehrband F Veale S Haidar A Ning L Dutta A. Perivascular space morphological changes in response to transcranial direct current stimulation. ISMRM Neuromodulation Workshop 2022. Available online at: https://www.ismrm.org/workshops/2022/Neuromodulation/program.php

[37] Pollock H Hutchings M Weller RO Zhang ET. Perivascular spaces in the basal ganglia of the human brain: their relationship to lacunes. J Anat. (1997) 191(Pt 3):337–46. 10.1046/j.1469-7580.1997.19130337.x 9418990 PMC1467691

[38] Khadka N Bikson M. Neurocapillary-modulation. Neuromodulation. (2022) 25:1299–311. 10.1111/ner.13338 33340187 PMC8213863

[39] Lang GE Vella D Waters SL Goriely A. Mathematical modelling of blood-brain barrier failure and oedema. Math Med Biol. (2017) 34:391–414. 10.1093/imammb/dqw009 27305934

[40] Friston KJ Fletcher P Josephs O Holmes A Rugg MD Turner R. Event-related fMRI: characterizing differential responses. Neuroimage. (1998) 7:30–40. 10.1006/nimg.1997.0306 9500830

[41] Arora Y Walia P Hayashibe M Muthalib M Chowdhury SR Perrey S. Grey-box modeling and hypothesis testing of functional near-infrared spectroscopy-based cerebrovascular reactivity to anodal high-definition tDCS in healthy humans. Res Sq [Preprint]. (2021). 10.21203/rs.3.rs-83907/v3 34613970 PMC8494321

[42] Lindquist MA Loh JM Atlas LY Wager TD. Modeling the hemodynamic response function in fMRI: efficiency, bias and mis-modeling. Neuroimage. (2009) 45:S187. 10.1016/j.neuroimage.2008.10.065 19084070 PMC3318970

[43] Boynton GM Engel SA Glover GH Heeger DJ. Linear systems analysis of functional magnetic resonance imaging in human V1. J Neurosci. (1996) 16:4207–21. 10.1523/JNEUROSCI.16-13-04207.1996 8753882 PMC6579007

[44] Ekhtiari H Ghobadi-Azbari P Thielscher A Antal A Li LM Shereen AD. A checklist for assessing the methodological quality of concurrent tES-fMRI studies (ContES checklist): a consensus study and statement. Nat Protoc. (2022) 17596–617. 10.1038/s41596-021-00664-5 35121855 PMC7612687

[45] Vincent T Badillo S Risser L Chaari L Bakhous C Forbes F. Flexible multivariate hemodynamics fMRI data analyses and simulations with PyHRF. Front Neurosci. (2014) 8:67. 10.3389/fnins.2014.00067 24782699 PMC3989728

[46] Nardo D Creasey M Negus C Pappa K Reid A Josephs O. Transcranial direct current stimulation with functional magnetic resonance imaging: a detailed validation and operational guide. (2021) 6:143. 10.12688/wellcomeopenres.16679.1 PMC10050906 37008187

[47] Wu G-R Colenbier N Van Den Bossche S Clauw K Johri A Tandon M. rsHRF: a toolbox for resting-state HRF estimation and deconvolution. Neuroimage. (2021) 244:118591. 10.1016/j.neuroimage.2021.118591 34560269

[48] Huang Y Datta A Bikson M Parra LC. Realistic volumetric-approach to simulate transcranial electric stimulation—ROAST—a fully automated open-source pipeline. J Neural Eng. (2019) 16:056006. 10.1088/1741-2552/ab208d 31071686 PMC7328433

[49] Jog MV Smith RX Jann K Dunn W Lafon B Truong D. *In-vivo* imaging of magnetic fields induced by transcranial direct current stimulation (tDCS) in human brain using MRI. Sci Rep. (2016) 6:34385. 10.1038/srep34385 27698358 PMC5048181

[50] Arora Y Chowdhury SR Dutta A. Physiological neurovascular modeling of cerebrovascular effects of transcranial electrical current stimulation. Brain Stimul. (2021) 14:1597–8. 10.1016/j.brs.2021.10.03134613970

[51] Guhathakurta D Dutta A. Computational pipeline for NIRS-EEG joint imaging of tDCS-evoked cerebral responses—an application in ischemic stroke. Front Neurosci. (2016) 10. 10.3389/fnins.2016.00261 27378836 PMC4913108

[52] Kaplan L Chow BW Gu C. Neuronal regulation of the blood–brain barrier and neurovascular coupling. Nat Rev Neurosci. (2020) 21:416. 10.1038/s41583-020-0322-2 32636528 PMC8934575

[53] Nitsche MA Bikson M. Extending the parameter range for tDCS: safety and tolerability of 4 mA stimulation. Brain Stimul. (2017) 10:541–2. 10.1016/j.brs.2017.03.002 28456325 PMC5972544

[54] Senatorov VV Friedman AR Milikovsky DZ Ofer J Saar-Ashkenazy R Charbash A. Blood-brain barrier dysfunction in aging induces hyperactivation of TGFβ signaling and chronic yet reversible neural dysfunction. Sci Transl Med. (2019) 11:eaaw8283. 10.1126/scitranslmed.aaw8283 31801886

[55] Wagshul ME Eide PK Madsen JR. The pulsating brain: a review of experimental and clinical studies of intracranial pulsatility. Fluids Barriers CNS. (2011) 8:5. 10.1186/2045-8118-8-5 21349153 PMC3042979

[56] Iliff JJ Wang M Zeppenfeld DM Venkataraman A Plog BA Liao Y. Cerebral arterial pulsation drives paravascular CSF–interstitial fluid exchange in the murine brain. J Neurosci. (2013) 33:18190. 10.1523/JNEUROSCI.1592-13.2013 24227727 PMC3866416

[57] Fonteneau C Mondino M Arns M Baeken C Bikson M Brunoni AR. Sham tDCS: a hidden source of variability? Reflections for further blinded, controlled trials. Brain Stimul. (2019) 12:668–73. 10.1016/j.brs.2018.12.977 30639235

[58] Sancho M Samson NC Hald BO Hashad AM Marrelli SP Brett SE. KIR channels tune electrical communication in cerebral arteries. J Cereb Blood Flow Metab. (2017) 37:2171–84. 10.1177/0271678X16662041 27466375 PMC5464710

[59] Dutta A Zhao F Cheung M Das A Tomita M Chatterjee K. Cerebral and muscle near-infrared spectroscopy during lower-limb muscle activity – volitional and neuromuscular electrical stimulation. In: 2021 43rd Annual International Conference of the IEEE Engineering in Medicine and Biology Society (EMBC). (2021) 6577–80. 10.1109/EMBC46164.2021.9629721 34892616

[60] von Bornstädt D Gertz K Lagumersindez Denis N Seners P Baron J-C Endres M. Sensory stimulation in acute stroke therapy. J Cereb Blood Flow Metab. (2018) 38:1682–9. 10.1177/0271678X18791073 30073883 PMC6168904

[61] Beishon LC Hosford P Gurung D Brassard P Minhas JS Robinson TG. The role of the autonomic nervous system in cerebral blood flow regulation in dementia: a review. Auton Neurosci. (2022) 240:102985. 10.1016/j.autneu.2022.102985 35525173

[62] Rezaee Z Dutta A. Transcranial direct current stimulation of the leg motor area - is it partly somatosensory? Annu Int Conf IEEE Eng Med Biol Soc. (2018) 2018:4764–7. 10.1109/EMBC.2018.8513195 30441414

[63] Glatte P Buchmann SJ Hijazi MM Illigens BM-W Siepmann T. Architecture of the cutaneous autonomic nervous system. Front Neurol. (2019) 10:970. 10.3389/fneur.2019.00970 31551921 PMC6746903

[64] Kistenmacher A Manneck S Wardzinski EK Martens JC Gohla G Melchert UH. Persistent blood glucose reduction upon repeated transcranial electric stimulation in men. Brain Stimul. (2017) 10:780–6. 10.1016/j.brs.2017.03.011 28392373

[65] Wardzinski EK Friedrichsen L Dannenberger S Kistenmacher A Melchert UH Jauch-Chara K. Double transcranial direct current stimulation of the brain increases cerebral energy levels and systemic glucose tolerance in men. J Neuroendocrinol. (2019) 31:e12688. 10.1111/jne.12688 30659676

[66] Szabadi E. Functional organization of the sympathetic pathways controlling the pupil: light-inhibited and light-stimulated pathways. Front Neurol. (2018) 9:1069. 10.3389/fneur.2018.01069 30619035 PMC6305320

[67] Imai J Katagiri H. Regulation of systemic metabolism by the autonomic nervous system consisting of afferent and efferent innervation. Int Immunol. (2022) 34:67–79. 10.1093/intimm/dxab023 33982088

[68] Johnson MS DeMarco VG Whaley-Connell A Sowers JR. Chapter 64 - Insulin resistance and the autonomic nervous system. In: Robertson D Biaggioni I Burnstock G Low PA Paton JFR editors. Primer on the Autonomic Nervous System, 3rd ed. San Diego, CA: Academic Press (2012), p. 307–12. 10.1016/B978-0-12-386525-0.00064-0

[69] Geddes JB Carr RT Wu F Lao Y Maher M. Blood flow in microvascular networks: a study in nonlinear biology. Chaos. (2010) 20:045123. 10.1063/1.3530122 21198135 PMC3026012

[70] He BJ. Scale-free properties of the functional magnetic resonance imaging signal during rest and task. J Neurosci. (2011) 31:13786–95. 10.1523/JNEUROSCI.2111-11.2011 21957241 PMC3197021

[71] Binkofski F Loebig M Jauch-Chara K Bergmann S Melchert UH Scholand-Engler HG. Brain energy consumption induced by electrical stimulation promotes systemic glucose uptake. Biol Psychiatry. (2011) 70:690–5. 10.1016/j.biopsych.2011.05.009 21703596

[72] Scholey AB Harper S Kennedy DO. Cognitive demand and blood glucose. Physiol Behav. (2001) 73:585–92. 10.1016/S0031-9384(01)00476-0 11495663

[73] Jolivet R Coggan JS Allaman I Magistretti PJ. Multi-timescale modeling of activity-dependent metabolic coupling in the neuron-glia-vasculature ensemble. PLoS Comput Biol. (2015) 11:e1004036. 10.1371/journal.pcbi.1004036 25719367 PMC4342167

[74] Sotero RC Trujillo-Barreto NJ. Modelling the role of excitatory and inhibitory neuronal activity in the generation of the BOLD signal. Neuroimage. (2007) 35:149–65. 10.1016/j.neuroimage.2006.10.027 17234435

[75] Sotero RC Trujillo-Barreto NJ. Biophysical model for integrating neuronal activity, EEG, fMRI and metabolism. Neuroimage. (2008) 39:290–309. 10.1016/j.neuroimage.2007.08.001 17919931

[76] Prichard J Rothman D Novotny E Petroff O Kuwabara T Avison M. Lactate rise detected by 1H NMR in human visual cortex during physiologic stimulation. Proc Natl Acad Sci USA. (1991) 88:5829–31. 10.1073/pnas.88.13.5829 2062861 PMC51971

[77] Ogawa S Tank DW Menon R Ellermann JM Kim SG Merkle H. Intrinsic signal changes accompanying sensory stimulation: functional brain mapping with magnetic resonance imaging. Proc Natl Acad Sci USA. (1992) 89:5951–5. 10.1073/pnas.89.13.5951 1631079 PMC402116

[78] Molaee-Ardekani B Márquez-Ruiz J Merlet I Leal-Campanario R Gruart A Sánchez-Campusano R. Effects of transcranial direct current stimulation (tDCS) on cortical activity: a computational modeling study. Brain Stimul. (2013) 6:25–39. 10.1016/j.brs.2011.12.006 22420944

[79] Attwell D Buchan AM Charpak S Lauritzen M MacVicar BA Newman EA. Glial and neuronal control of brain blood flow. Nature. (2010) 468:232–43. 10.1038/nature09613 21068832 PMC3206737

[80] Farr H David T. Models of neurovascular coupling *via* potassium and EET signalling. J Theor Biol. (2011) 286:13–23. 10.1016/j.jtbi.2011.07.006 21781976

[81] Kuschinsky W Wahl M Bosse O Thurau K. Perivascular potassium and pH as determinants of local pial arterial diameter in cats. A microapplication study. Circ Res. (1972) 31:240–7. 10.1161/01.RES.31.2.240 5049739

[82] Bellot-Saez A Kékesi O Morley JW Buskila Y. Astrocytic modulation of neuronal excitability through K+ spatial buffering. Neurosci Biobehav Rev. (2017) 77:87–97. 10.1016/j.neubiorev.2017.03.002 28279812

[83] Gu X Chen W Volkow ND Koretsky AP Du C Pan Y. Synchronized astrocytic Ca^2+^ responses in neurovascular coupling during somatosensory stimulation and for the resting state. Cell Rep. (2018) 23:3878–90. 10.1016/j.celrep.2018.05.091 29949771 PMC7469112

[84] Longden TA Hill-Eubanks DC Nelson MT. Ion channel networks in the control of cerebral blood flow. J Cereb Blood Flow Metab. (2016) 36:492–512. 10.1177/0271678X15616138 26661232 PMC4794103

[85] Rojas M Chávez-Castillo M Bautista J Ortega Á Nava M Salazar J. Alzheimer's disease and type 2 diabetes mellitus: pathophysiologic and pharmacotherapeutics links. World J Diabetes. (2021) 12:745–66. 10.4239/wjd.v12.i6.745 34168725 PMC8192246

[86] Kandimalla R Thirumala V Reddy PH. Is Alzheimer's disease a Type 3 diabetes? A critical appraisal. Biochim Biophys Acta Mol Basis Dis. (2017) 1863:1078–89. 10.1016/j.bbadis.2016.08.018 27567931 PMC5344773

[87] Canna A Esposito F Tedeschi G Trojsi F Passaniti C di Meo I. Neurovascular coupling in patients with type 2 diabetes mellitus. Front Aging Neurosci. (2022) 14:976340. 10.3389/fnagi.2022.976340 36118711 PMC9476313

[88] Kuehn BM. In Alzheimer research, glucose metabolism moves to center stage. JAMA. (2020) 323:297–9. 10.1001/jama.2019.20939 31913419

[89] Singh VP Bali A Singh N Jaggi AS. Advanced glycation end products and diabetic complications. Korean J Physiol Pharmacol. (2014) 18:1–14. 10.4196/kjpp.2014.18.1.1 24634591 PMC3951818

[90] Meisl G Hidari E Allinson K Rittman T DeVos SL Sanchez JS. *In vivo* rate-determining steps of tau seed accumulation in Alzheimer's disease. Sci Adv. (2021) 7:eabh1448. 10.1126/sciadv.abh1448 34714685 PMC8555892

[91] Kisler K Nelson AR Rege SV Ramanathan A Wang Y Ahuja A. Pericyte degeneration leads to neurovascular uncoupling and limits oxygen supply to brain. Nat Neurosci. (2017) 20:406–16. 10.1038/nn.4489 28135240 PMC5323291

[92] Fowler MJ. Microvascular and macrovascular complications of diabetes. Clin Diabetes. (2008) 26:77–82. 10.2337/diaclin.26.2.77

[93] Rouch L Cestac P Sallerin B Andrieu S Bailly H Beunardeau M. Pulse wave velocity is associated with greater risk of dementia in mild cognitive impairment patients. Hypertension. (2018) 72:1109–16. 10.1097/01.hjh.0000549388.13724.61 30354804

[94] Ovadia-Caro S Khalil AA Sehm B Villringer A Nikulin VV Nazarova M. Predicting the response to non-invasive brain stimulation in stroke. Front Neurol. (2019) 10:302. 10.3389/fneur.2019.00302 31001190 PMC6454031

[95] Kaur J Fahmy LM Davoodi-Bojd E Zhang L Ding G Hu J. Waste clearance in the brain. Front Neuroanat. (2021) 15:53. 10.3389/fnana.2021.665803 34305538 PMC8292771

[96] Barbiellini Amidei C Fayosse A Dumurgier J Machado-Fragua MD Tabak AG van Sloten T. association between age at diabetes onset and subsequent risk of dementia. JAMA. (2021) 325:1640–9. 10.1001/jama.2021.4001 33904867 PMC8080220

[97] Nikulin VV Fedele T Mehnert J Lipp A Noack C Steinbrink J. Monochromatic ultra-slow (~0.1 Hz) oscillations in the human electroencephalogram and their relation to hemodynamics. Neuroimage. (2014) 97:71–80. 10.1016/j.neuroimage.2014.04.008 24732648

[98] Zhao F Tomita MR Dutta A. Functional near-infrared spectroscopy of prefrontal cortex during memory encoding and recall in elderly with type 2 diabetes mellitus. Annu Int Conf IEEE Eng Med Biol Soc. (2022) 2022:3323–6. 10.1109/EMBC48229.2022.9871983 36086207

[99] Kim Y-K Nam KI Song J. The glymphatic system in diabetes-induced dementia. Front Neurol. (2018) 9:867. 10.3389/fneur.2018.00867 30429819 PMC6220044

[100] Jindal U Sood M Dutta A Chowdhury SR. Development of point of care testing device for neurovascular coupling from simultaneous recording of EEG and NIRS during anodal transcranial direct current stimulation. IEEE J Transl Eng Health Med. (2015) 3:2000112. 10.1109/JTEHM.2015.2389230 27170897 PMC4848058

[101] Wiehler A Branzoli F Adanyeguh I Mochel F Pessiglione M. A neuro-metabolic account of why daylong cognitive work alters the control of economic decisions. Curr Biol. (2022) 32:3564–75.e5. 10.1016/j.cub.2022.07.010 35961314

