# Supplementary material for: Perspective: Disentangling the effects of tES on neurovascular unit
Source: Front Neurol. 2023 Jan 9;13:1038700. doi: 10.3389/fneur.2022.1038700 (PMC9868757; doi:10.3389/fneur.2022.1038700)
Supplement: Supplementary file 1 [file Data_Sheet_1.pdf]

<u>**Supplementary Material**</u>

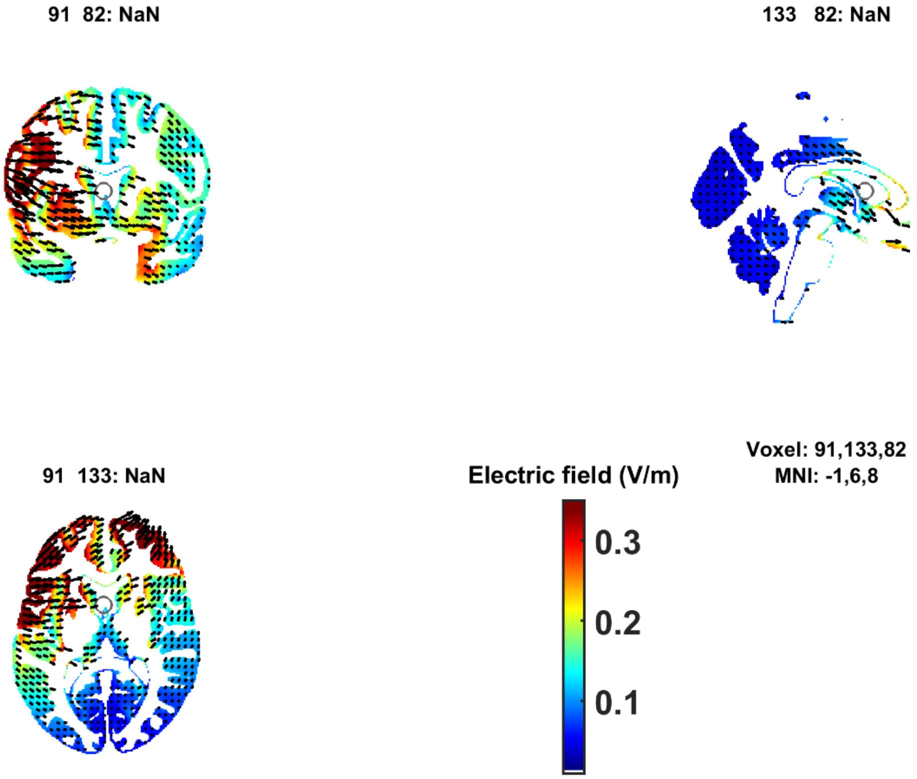

(A)

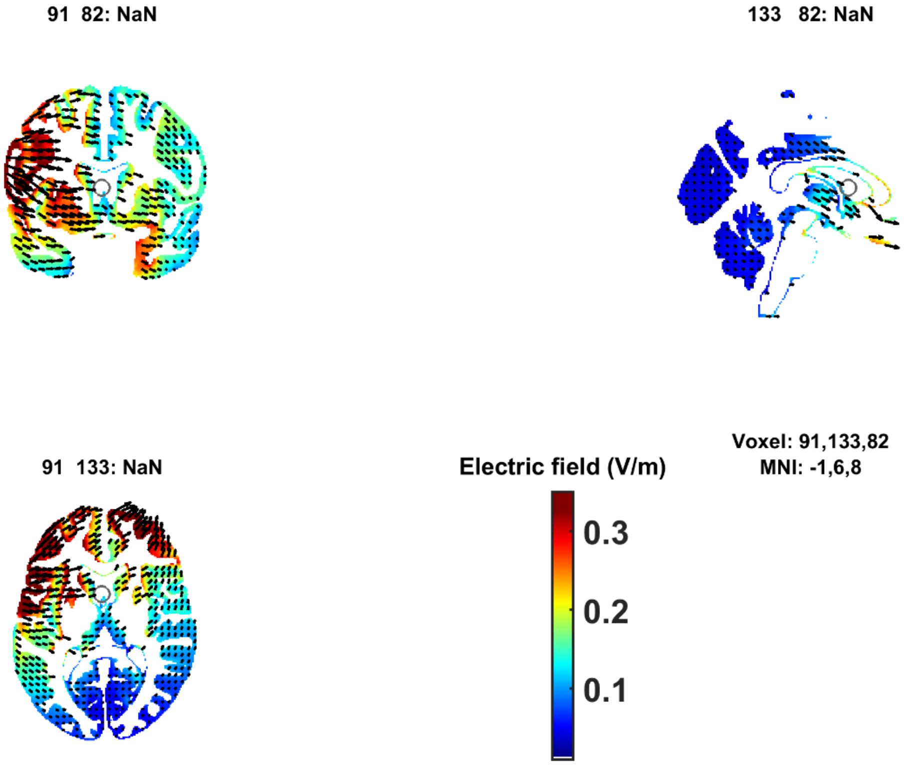

(B)

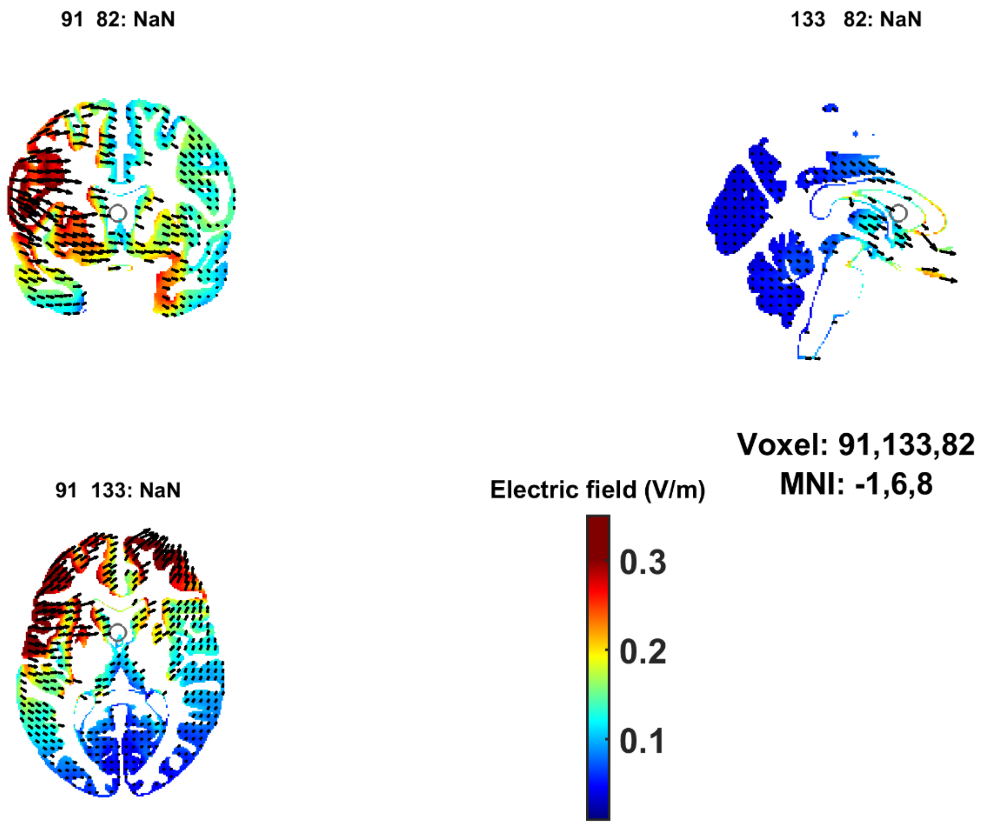

(C)

Figure S1: Electrical Field distribution in the gray matter of the brain for 2mA tDCS with FC5 (anodal electrode) and FP2 (cathodal electrode) in ROAST using default MNI152 head (https://github.com/andypotatohy/roast). An increase in the conductivity of the gray matter and the white matter is postulated due to tES-evoked increase in the blood volume fraction in the brain tissue where blood has a higher average isotropic conductivity than white matter and gray matter https://itis.swiss/virtual-population/tissue-properties/database/low-frequency-conductivity/. Default conductivities in ROAST (https://github.com/andypotatohy/roast), cerebrospinal fluid (default 1.65 S/m); bone (default 0.01 S/m); skin (default 0.465 S/m); air (default 2.5e-14 S/m); gel (default 0.3 S/m); electrode (default 5.9e7 S/m). (A) white matter (default 0.126 S/m); gray matter (default 0.276 S/m); (B) white matter (default 0.126 S/m); gray matter (0.3 S/m); (C) white matter (0.1575 S/m); gray matter (0.345 S/m). https://doi.org/10.1016/j.neuroimage.2004.09.043
